# Age-related guanine nucleotide exchange factor, mouse Zizimin2, induces filopodia in bone marrow-derived dendritic cells

**DOI:** 10.1186/1742-4933-9-2

**Published:** 2012-04-11

**Authors:** Isamu Sakabe, Azusa Asai, Junko Iijima, Mitsuo Maruyama

**Affiliations:** 1 Department of Mechanism of Aging, Research Institute - National Center for Geriatrics and Gerontology, 35, Gengo, Morioka-Machi, Obu-city, Aichi 474-8511, Japan

**Keywords:** GEF, Zizimin2, Filopodia, Cdc42⋅ Dendritic cells

## Abstract

**Background:**

We recently isolated and identified Zizimin2 as a functional factor that is highly expressed in murine splenic germinal center B cells after immunization with T-cell-dependent antigen. Zizimin2 was revealed to be a new family member of Dock (dedicator of cytokinesis), Dock11, which is the guanine nucleotide exchange factor for Cdc42, a low-molecular-weight GTPase. However, the molecular function of Zizimin2 in acquired immunity has not been elucidated.

**Results:**

In this study, we show that the protein expression of Zizimin2, which is also restricted to lymphoid tissues and lymphocytes, is reduced in aged mice. Over-expression of full-length Zizimin2 induced filopodial formation in 293T cells, whereas expression of CZH2 domain inhibited it. Stimulation of Fcγ receptor and Toll-like receptor 4 triggered Zizimin2 up-regulation and Cdc42 activation in bone marrow-derived dendritic cells.

**Conclusions:**

These data suggest that Zizimin2 is an immune-related and age-regulated guanine nucleotide exchange factor, which facilitates filopodial formation through activation of Cdc42, which results in activation of cell migration.

## Background

Zizimin2, also called Dock11 (dedicator of cytokinesis 11), has been reported as one of the human Dock180 super-family proteins [1]. We recently identified Zizimin2 as a 238-kDa protein that is highly expressed in germinal center B lymphocytes after T-cell-dependent antigen immunization [2]. The expression of this gene is regulated in lymphocytes and organs, such as spleen, thymus, and lymph nodes [2,3]. It has been shown that Zizimin2 binds and activates nucleotide-free Cdc42 via its CZH2 [CDM(ced5/DOCK180/myoblast city)-Zizimin homology 2] domain [4] and mediates positive feedback on the active form, GTP-bound Cdc42 [5]. Cdc42, one of the well-known Rho family members, regulates signaling pathways that control diverse cellular functions including morphology, migration, endocytosis, and cell cycle progression [6-8]. These reports suggest that Zizimin2 has a role in regulating the pathway downstream of Cdc42, leading to cellular functions.

Cdc42 affects the formation of highly dynamic finger-like actin-rich protrusions known as filopodia. Filopodia contain parallel bundles of filamentous F-actin, and are thought to be important for sensing the environment, for example, guidance toward chemoattractants [9,10]. Cdc42 induces filopodia and lamellipodia in activated B cells [11]. Microinjection of constitutively active Cdc42 stimulates filopodial extension in immature dendritic cells [12]. Another isoform of Zizimin subfamily, Zizimin1 (Dock9) is also known to be capable to interact with Cdc42 through the CZH2 domain, and this interaction induces the formation of filopodia in NIH-3T3 cells expressing exogenous murine Zizimin1, although their tissue distribution is remarkably different [2,13]. DOCK2-deficient plasmacytoid dendritic cells (pDCs) failed to migrate into the periarteriolar lymphoid sheaths of the spleen [14]. In DOCK-deficient pDCs, chemokine-induced Rac activation was severely impaired, resulting in the reduction of motility and the loss of polarity during chemotaxis [14].

Although upstream effectors for Cdc42, which activates the pathway to filopodial formation, especially receptors located in plasma membrane, have not been identified yet, Fc receptor and Toll-like receptor (TLR) are candidates for the receptors because they are involved in many immune responses. Fc receptor is a protein found on the surface of certain cells, including natural killer cells, macrophages, neutrophils, dendritic cells, and mast cells, and contributes to the protective functions of not only the innate but also acquired immune system. There are several different types of Fc receptor, which are classified on the basis of the type of antibody that they recognize. For example, Fcγ receptors (FcγR) recognize IgG. They interact with an IgG-coated antigen so they are involved in acquired immunity [15]. TLR belongs to the family of pattern recognition receptors that are used to recognize microbial products from several classes of microbes, as well as endogenous ligands. TLR4 is a receptor for gram-negative lipopolysaccharide (LPS), respiratory syncytial virus protein F, and other endogenous ligands, such as surfactant protein A and fibronectin fragment [16].

These findings led us to clarify Zizimin2 function regarding the signaling pathway leading to cell migration. In this study, we show that Zizimin2 facilitates filopodial formation via activation of Cdc42 as a downstream effector of Fcγ receptor or TLR4 in bone marrow-derived dendritic cells (BMDC) and that Zizimin2 CZH2 domain has dominant negative effects on cell migration.

## Results

### Zizimin2 is expressed in lymphoid organs

**Figure 1:**
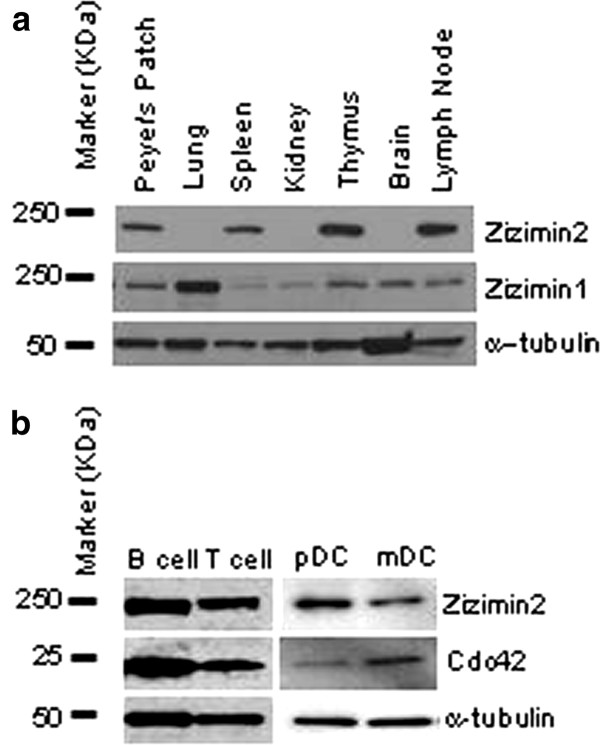
**Expression profile of Zizimin1 and Zizimin2 protein**. **A) **Western blot analysis shows the expression levels of Zizimin1 and Zizimin2 in murine tissues described at the top of the panels. α-Tubulin was used as a loading control. **B) **Western blot analysis of Zizimin2 was carried out with cell extracts from splenic B cells, splenic T cells, and pDC or mDC from bone marrow. α-Tubulin was used as a loading control.

Since Zizimin2 was identified as a gene that is highly expressed in murine splenic germinal center B cells after immunization with T-cell-dependent antigen, and we recently developed rat monoclonal anti-mouse Zizimin2 antibody [3], we first confirmed the expression level of Zizimin2 protein in murine tissues. Western blot showed that Zizimin2 is highly expressed in the lymphoid organs such as thymus, spleen, lymph node (Figure 1A), splenic B cells, T cells, myeloid DCs, and pDCs (Figure 1B). An equal amount of protein loading was confirmed by pomso staining of the membrane (Additional file 1: Figure S1).

### Zizimin2 is down-regulated in aged mice

**Figure 2:**
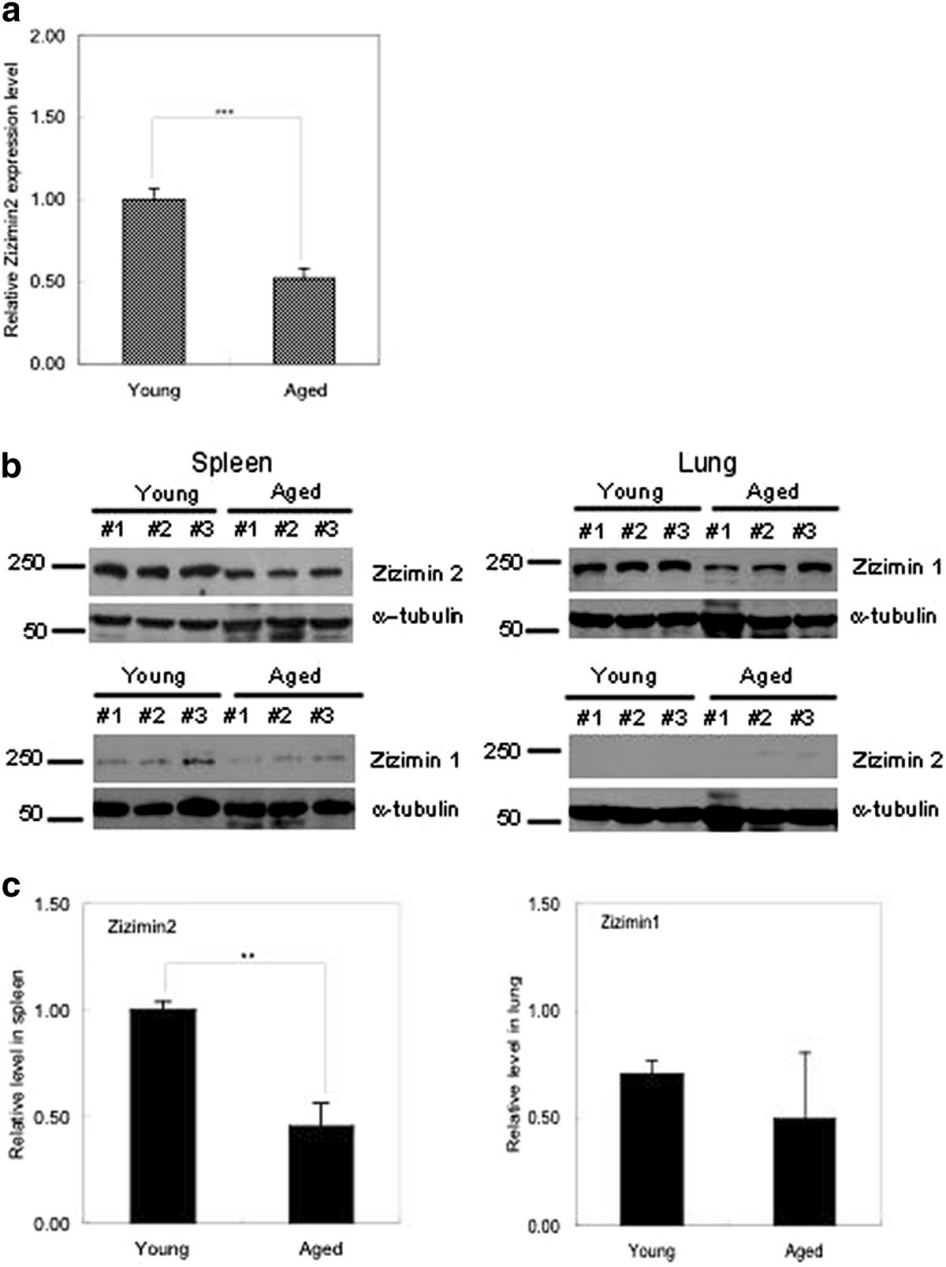
**Zizimin2, Zizimin1, and Zizimin3 expression in young and aged mice**. **A) **Total RNA was prepared from young (7-8 weeks old) or aged (24 months old) mice, followed by reverse transcription to obtain the cDNA. Quantitative RT-PCR was carried out with specific primer sets for Zizimin2 and G3PDH. Relative mRNA levels were calculated using the delta-delta CT method using G3PDH as a housekeeping gene. Asterisks indicate that the sample group is significantly different from aged mice at *** *P *< 0.0001 by Student's *t*-test. Means ± S.D. values of four experiments are presented. **B) **Lysates were prepared from three individual three young (7-8 weeks old) or aged (24 months old) mouse tissues, which are described to the left of each panel, followed by western blotting. α-Tubulin was used as a loading control. **C) **Expression levels of Zizimin2 and Zizimin1 were quantified as relative levels. Means ± S.D. values of three experiments are presented. ** *P *< 0.01 by Student's *t*-test.

A correlational evidences between aging and immunosenescence has been intensively debated for a decade [17-19], but the detailed mechanism of immunosenescence has not been revealed yet. Therefore, the issue of the expression of Zizimin2 being regulated in an age-dependent manner has been our fllowing prominent interest. Quantitative RT-PCR analysis shows that Zizimin2 gene is distinctly down-regulated in aged mouse spleen (Figure 2A). Moreover, the expression level of Zizimin2 protein in aged mouse spleen was less than that in young mouse spleen (7-8 weeks old); on the other hand, the expression level of Zizimin1 in lung was stable, suggesting that Zizimin2 expression is saliently regulated in age-dependent manner in mouse spleen (Figure 2B, C).

### Induction of filopodia

**Figure 3:**
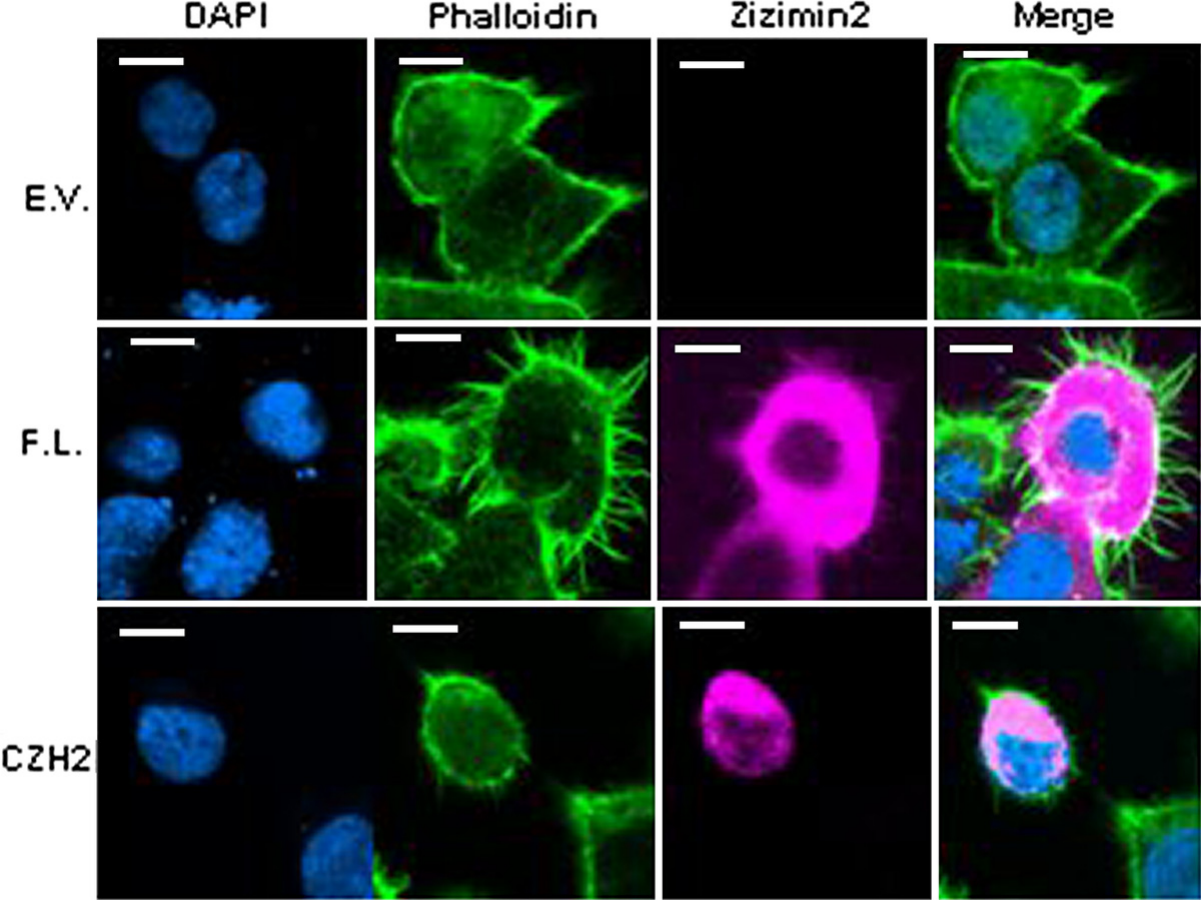
**Over-expression of Zizimin2 induced filopodial formation**. 293 T cells transfected with empty vector, or expression vectors with full-length Zizimin2 or its CZH2 domain (CZH2) were stained with DAPI (DAPI), phalloidin (phalloidin), and anti-Zizimin2 antibody (Zizimin2) followed by immunofluorescence microscopy. Right panels are merged images of phalloidin-stained and anti-Zizimin2-stained images. White bars are 10 μm.

It has been shown that Cdc42 is a signal transducer in the pathway leading to the induction of filopodial formation [9] and Zizimin2 interacts with and activates Cdc42 [2]. Therefore, it is speculated that over-expression of Zizimin2 induces filopodial formation. In order to confirm this, Cdc42 or Zizimin2 was over-expressed in 293T cells followed by immunofluorescence microscopy. Over-expression of Cdc42WT and its active form (Cdc42V12) in 293T cells induced filopodia, whereas the negative form (Cdc42N17) did not inhibit them, suggesting that Cdc42 is a positive regulator of filopodial formation (Additional file 2: Figure S2). Over-expression of full-length Zizimin2 induced filopodia, whereas CZH2 domain prevented this induction, suggesting that Zizimin2 is also a positive regulator of filopodial formation, which plays important physiological roles in guidance toward chemoattraction (Figure 3) [10].

### Zizimin2 is activated through Fcγ receptor II/III or TLR4 signaling pathway

**Figure 4:**
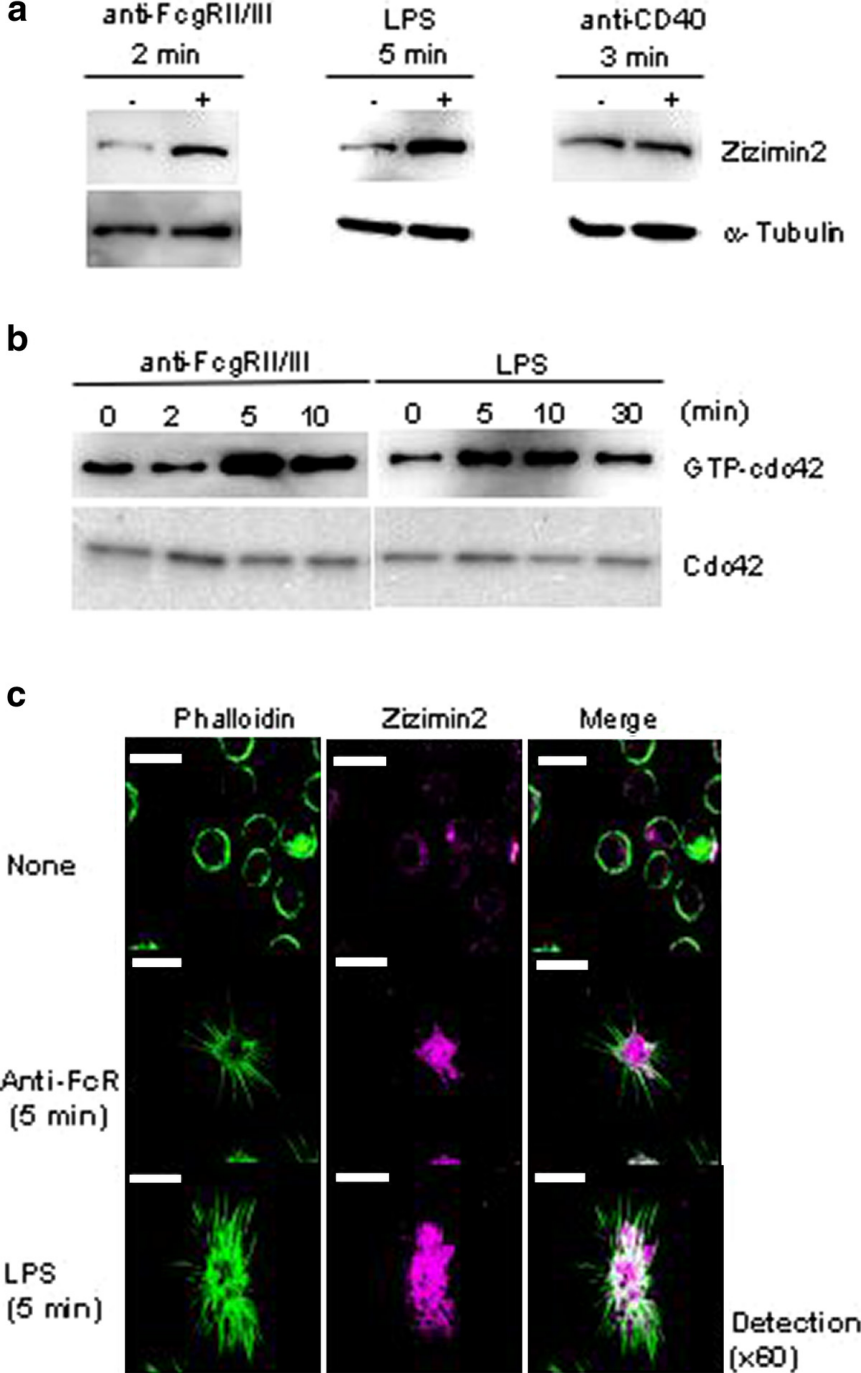
**A) Up-regulation of Zizimin2 in response to stimulations**. 10^7^cells/ml of BMDC without or with stimulation by 1 μg/ml anti-IgG Fab'2 for 2 min (left panel, anti-FcγRII/III), 1 μg/ml LPS for 5 min (middle panel), or 1 μg/ml anti-CD40 for 3 min (right panel) were lysed and subjected to western blotting. α-Tubulin was used as a loading control. **B) **Cdc42 activation in response to stimulations. 10^7^cells/ml of BMDC were stimulated with 1 μg/ml anti-IgG Fab'2 for 0 to 10 min (left panel, anti-FcγRII/III) or 1 μg/ml LPS for 0 to 30 min (right panel) and subjected to Cdc42 activation assay. Activated Cdc42 was assayed by precipitating GTP-loaded Cdc42 with bead-bound GST-PAK1-PBD, and detection of Cdc42 in pull downs (upper panel) or total cell lysates (lower panel) by western blotting with anti-Cdc42. **C) **Filopodial formation in BMDC. BMDC without or with stimulation by 1 μg/ml anti-IgG Fab'2 for 5 min (anti-FcγR) or 1 μg/ml LPS for 5 min were stained with phalloidin, which was bound with F-actin (left panel, green), anti-mouse-Zizimin2 antibody, and anti-rat-IgG-Cy3-conjugated antibody (middle panel, magenta) followed by immunofluorescence microscopy. Right panels are merged images of detected phalloidin (Alexa488) and detected Zizimin2 (Cy3). White bars are 100 μm.

We reported that Zizimin2 binds and activates nucleotide-free Cdc42 via its CZH2 domain [2]. To gain further insight into the intracellular signaling of Zizimin2, we investigated whether Fcγ receptors, TLR4 and CD40, from those which signaling is important for the immune response of dendritic cells [16,20,21], are associated with Zizimin2 or Cdc42. Stimulation of BMDC with anti-Fcγ receptor II/III or LPS, which is a TLR4 ligand, induced Zizimin2 up-regulation and Cdc42 activation, which was indicated by an increase in the ratio of GTP-bound Cdc42 versus total Cdc42 (Figure 4A, B). On the other hand, CD40 did not affect Zizimin2 expression, therefore, Zizimin2 does not seem to play a role in the CD40 signaling pathway (Figure 4A).

We further investigated whether activation of the Fcγ receptor II/III or TLR4 induced filopodia. The stimulated BMDC with anti-Fcγ receptor II/III or LPS induced filopodia, which were shown by protrusions stained with phalloidin (green) and partially merged with Zizimin2 expression sites (magenta), shown as white regions, and up-regulation of Zizimin2 expression (Figure 4C). These results suggest that Cdc42 and Zizimin2 play roles in filopodial formation as signal transducers from Fcγ receptor II/III and TLR4 in dendritic cells.

## Discussion

### Tissue distribution of Zizimin subfamily

According to their sequence similarity, Zizimin subfamily includes three genes named Zizimin1/DOCK9, Zizimin2/DOCK11 and Zizimin3/DOCK10 [1,13]. Zizimin2 is expressed predominantly in hematopoietic tissues, especially, lymphoid organs for both acquired and innate immune resoponse. Zizimin1 is predominantly involved in non-hematopoietic tissues (Figure 1A), and Zizimin3 is expressed in human peripheral blood B lymphocytes involved in IL-4 signaling pathway [22]. Taken together the specificity of Zizimin family, we suggest that Zizimin2 engages in mouse immune-system-specific signaling pathway.

### Age dependence of Zizimin2 expression

The expression level of Zizimin2 decreased in aged mice compared with that in young mice, (Figure 2A-C), which strongly suggests that the expression of Zizimin2 might be regulated by an age-dependent mechanism. The decreased expression might contribute to physiological defects that are seen in the aged body, especially related to immunosenescence.

### Function of CZH2 domain

We examined and revealed that CZH2 domain of Zizimin2 inhibited filopodial formation followed by cell migration, which indicated that the CZH2 domain possesses dominant negative effects on cell migration (Figure 3). It is speculated that the CZH2 domain in Zizimin1 is inevitable for interaction with Cdc42 and its own PH domain [23,24]. In cases where Cdc42 is at a low level, its CZH2 domain interact is capable to with PH domain, namely as a closed form, so Zizimin1 might be resting status for the signaling pathway. On the other hand, in cases where active Cdc42 is abundant, the CZH2 domain is released from the PH domain, as a open form. The open form can interact with Cdc42 and activate it. This information surely lends further support to notion that Zizimin2 also plays a physiological role for lymphocyte migration with its PH and CZH2 domains.

### Mechanism of Zizimin2 up-regulation by stimulation

We describe here Cdc42 activation and Zizimin2 up-regulation in response to anti-Fcγ receptor II/III or LPS stimulation in BMDC (Figure 4A, B). It is worth nothing that the response of Zizimin2 was so rapid that regulation at the transcription or translation step might not be involved in this up-regulation. Therefore, we speculate that inhibition of Zizimin2 protein degradation is one of the mechanisms to explain the rapid response. However, as shown in Additional file 3: Figure S3 more Zizimin2 protein was detected in the cells stimulated with anti-Fcγ receptor II/III or LPS than the cells treated with MG132, which is a proteasome inhibitor. Although further analysis should be required, this result allows us to infer that there is another mechanism to increase Zizimin2 protein in the stimulated cells.

### Zizimin2-mediated signaling pathway for lymphocyte migration

**Figure 5:**
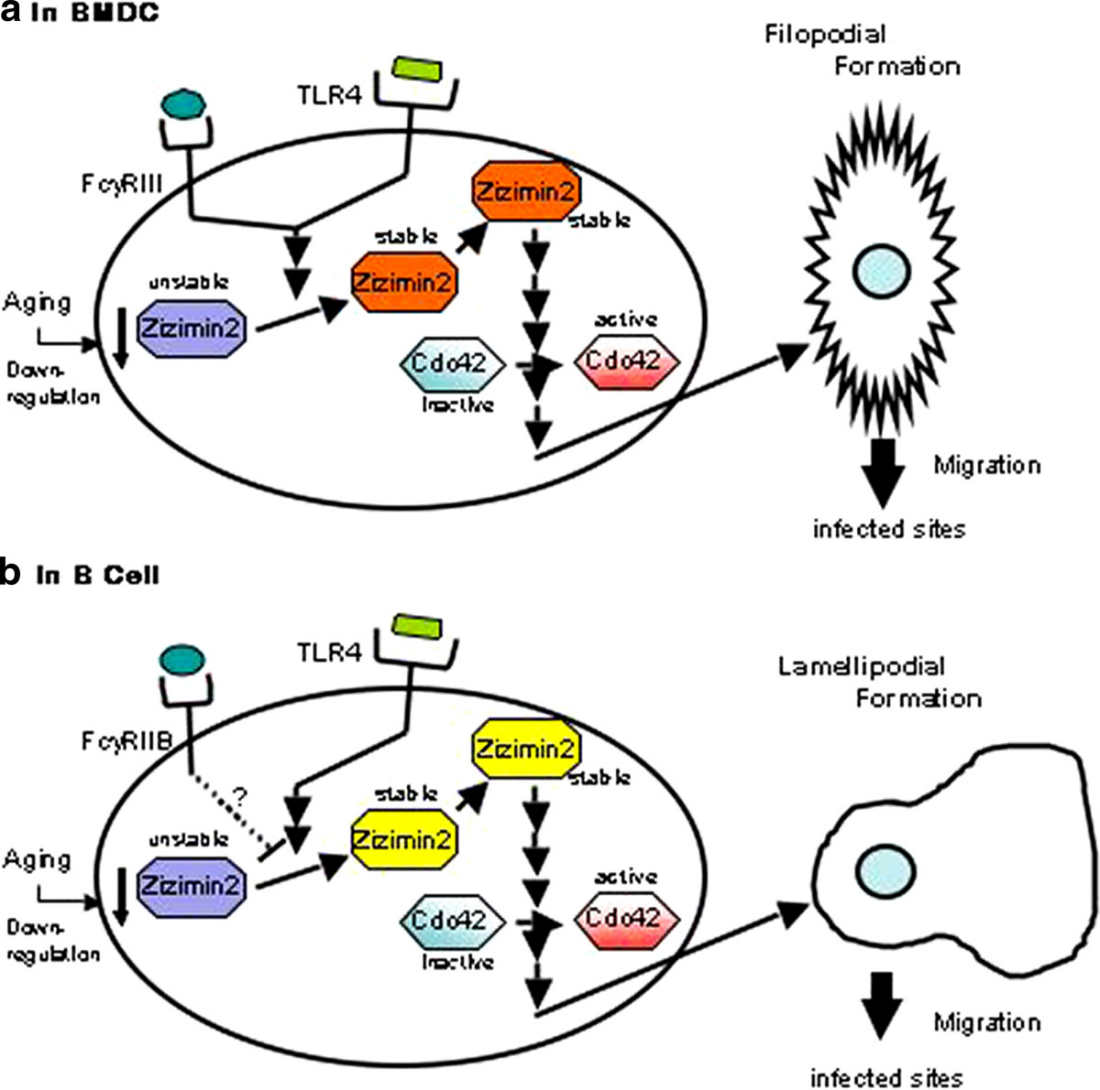
**Schematic representation of putative lymphocyte migration pathway through Zizimin2**. **A) **In BMDC, constitutively expressed FcγRIII or TLR4 is activated by each ligand, resulting in the activation of Zizimin2 with the conversion from its unstable to stable form. Then the Zizimin2 interacts with plasma membrane through its own PH domain and activates Cdc42, followed by activation of the downstream effectors, consequently induces filopodial formation for migration to sites the pathogens infected. Significantly, aging induces down-regulation of Zizimin2 expression, resulting in reduction of filopodial formation. **B) **In B cells, for TLR4 signaling is cardinally active as in BMDC might be caused, except eventually lamellipodial formation. It might be capable for FcγRIIB to transduce a inhibitory function against Zizimin2 while the signal from TLR4 comes out.

Taken together with our present results of the signaling pathway for filopodial formation with Zizimin2, we proposed a signaling pathways for lymphocyte migration depicted in Figure 5. In BMDC, once receiving the signals from the cognate ligands, both FcγRIII and TLR4 induce and stabilize Zizmin2 as a stable form capable to be recruited to plasma membrane through its own PH domain and activates Cdc42, followed by activation of the downstream effectors, eventually it reaches to filopodial formation for migration to sites pathogens infected. As aging induces down-regulation of Zizimin2 expression, reduction of filopodial formation and migration might be observed in aged mice.

As demonstrated in Figure 1B, Zizimin2 expression has been observed in splenic B and T lymphocytes as well as BMDC. Therefore, we also speculate the same events as in BMDC regarding from TLR4 might be caused in these lymphocytes. Since it has been reported not filopodial but lamellipodial formation plays an important role in migration of B cells and T cells, through Cdc42 [25,26]. Fothermore, FcγRIIB is known only FcγR which expresses in B cells and transduces a inhibitory signal for immunoglobulin production [27,28], this information lends further support to speculate that FcγRIIB also transduce a inhibitory signal against Zizimin2 regardless of TLR4 signaling. In addition to the verification of these Zizimin2 mediated signaling pathway, further understanding of physiological function of Zizimin2 in immune system should provide invaluable insights into improvements in immune scenesense related to infection and immunity.

## Conclusions

These data suggest that Zizimin2 is a novel immune-system-related and age-regulated guanine nucleotide exchange factor, which accelerates filopodial formation through activation of Cdc42 in response to extracellular stimulation, which leads to activation of lymphocyte migration.

## Methods

### Cell culture and transfection

293T cells were grown in Dulbecco's modified Eagle's Medium (DMEM, Wako Pure Chemical Industries, Osaka, Japan) supplemented with 10% fetal bovine serum (FBS, EQUITECH-BIO, Ingram, TX). For transfection, 293T cells in 6-well tissue-culture plates at approximately 70% confluence were transfected with a total of 1 μg of plasmid vector DNA using the Fugene 6 transfection reagent (Roche, Mannheim, Germany) according to the manufacturer's instructions.

### DNA constructs

Constructs described below were used for over-expression of full-length Zizimin2 or its CZH2 domain. DNA coding full-length Zizimin2 or its CZH2 domain was cloned into pCNX2-HA using NotI site for full-length or EcoRI and XhoI for its CZH2 domain, which are referred to as HA-Zizimin2 expression vector, pCNX2-HA-Zizimin2, and HA-CZH2 domain expression vector, pCNX2-HA-CZH2.

### Immunoblotting analysis

Tissues or cells were lysed by the addition of RIPA buffer (25 mM Tris-HCl pH 7.6, 150 mM NaCl, 1 mM EDTA, 1% NP-40, 1% sodium deoxycholate, 0.1% SDS) with protease inhibitors (Roche, Mannheim, Germany) and homogenized on ice with a homogenizer. Insoluble materials were pelleted by centrifugation at 15,000 g for 20 min at 4°C, and protein in the supernatant was quantified by bicinchoninic acid protein assay (Pierce, Rockford, IL). Protein dissolved in SDS sample loading buffer (62.5 mM Tris-HCl (pH6.8), 5% 2-mercaptoethanol, 2% SDS, 5% sucrose, 0.002% bromophenol blue) was boiled for 5 min, subjected to SDS-PAGE using 5-20% or 8% polyacrylamide gel, and transferred to polyvinylidene fluoride membrane. Membrane was blocked in PBS containing 0.1% Tween-20 (PBST) and 1% non-fat milk for Zizimin1 and Zizimin2 or 5% non-fat milk for α-tubulin overnight at 4°C. Then, the membrane was probed for 2 h with Zizimin2 antibody (1:100) (214I9, [3]), Zizimin1 antibody (1:1000) (NB500-265, Novus Biologicals, Littleton, CO), and anti-α-tubulin antibody (1:2000) (Sigma-Aldrich, St. Louis, MO) in PBST containing 1% non-fat milk, washed three times with PBST, and incubated with peroxidase-conjugated secondary antibody (1:2000) (Jackson ImmunoResearch, West Grove, PA) for 2 h at room temperature (RT). After several washes with PBST, reaction was visualized with Immobilon Western (MILLPORE, Billerica, MA) for detection of Zizimin2, Zizimin1, and Cdc42 or ECL (ECL plus Western blotting Detection System, GE Healthcare, Piscataway, NJ) for α-tubulin.

### Quantitative real-time RT-PCR

Quantitative real-time RT-PCR was carried out as described previously [29]. The following gene-specific primers were used: mouse Zizimin2, 5'-TTG CCT TTT ATG GCC AGT CT-3' (sense) and 5'-GAG CGA ATT TTG GAT CAA GC-3' (antisense), mouse GAPDH, 5'-AAT GGT GAA GGT CGG TGT G-3' (sense) and 5'-GAA GAT GGT GAT GGG CTT CC-3' (antisense).

### Cdc42 activation assay

For Cdc42 activation assay, we used the Cdc42 activation kit (Cytoskeleton, Denver, CO) according to the manufacturer's instructions.

### Immunofluorescence microscopy

293T cells and BMDC cultured on poly-lysine-coated coverslips were washed three times in PBS, fixed for 15 min in 4% paraformaldehyde at 37°C, washed in PBS, and permeabilized with 0.1% TritonX-100 in PBS for 5 min at RT. The coverslips were washed three times in PBS, blocked for 1 h with 0.2% bovine serum albumin in PBS, washed three times in PBS, and incubated for 1 h with 214I9 hybridoma culture supernatant (1:1) or 214I9 (1:100) [3] for Zizimin2 staining, Alexa488-conjugated phalloidin (1:500) (Invitrogen, Carlsbad, CA) for actin staining, or sheep anti-cdc42 (1:100) (Cytoskeleton, Denver, CO) for Cdc42 staining. Following three washes in PBS, coverslips were incubated for 1 h with Cy3-labeled secondary antibodies (Jackson ImmunoResearch, West Grove, PA), washed three times in PBS, and mounted onto microscope slides using Vectashield containing 4',6-diamino-2-phenylindole (DAPI, Vector Labs, Burlingame, CA) for nucleus detection. Images were acquired using a Fluoview laser scanning microscope (OLYMPUS, Tokyo, Japan).

### Isolation of BMDC, pDC, and mDC

Bone marrow cells obtained from C57/BL6 mouse were maintained in RPMI1640 supplemented with 10% X63-GM cell culture supernatants as a source of granulocyte macrophage colony-stimulating factor (GM-CSF) or 50 ng/ml FMS-like tyrosine kinase 3 (Flt3) (R&D SYSTEMS, Minneapolis, MN) for 6 days to induce BMDC. On the 6th day, the cells were stimulated with 1 μg/ml lipopolysaccharide (LPS) and cultured for 2 more days. On the 8th day, the cells were stained with FITC-conjugated anti-CD11c antibody (BD Bioscience, Franklin Lakes, NJ) and CD11c-positive cells were sorted using a cell sorter, JSAN (Bay Bioscience, Kobe, Japan), which were defined as BMDC in this study. pDC (CD11c^+^, B220^+^, mPDCA-1^+^, Ly-6C^+^) and mDC (CD11c^+^, B220^-^) were sorted from BMDC using a cell sorter, JSAN (Bay Bioscience, Kobe, Japan). All animal procedures were approved by institutional reviews board at National Center for Geriatrics and Gerontology followed the guideline issued by Japanese Ministry of Health Labour and Welfare.

### Isolation of B cells and T cells

Splenocyte suspension was prepared from mouse spleen and splenic B cells or T cells were isolated with MACS beads (Miltenyi Biotec, Gladbach, Germany), according to the manufacturer's instructions.

## Competing interests

The authors declare that they have no competing interests.

## Authors' contributions

Isamu Sakabe carried out isolation of B cells and T cells, western blot shown in Figure 1B, immunofluorescence microscopy shown in Figure 3A and Additional file 2: Figure S2, and drafted the manuscript. Azusa Asai designed the study and performed preparation of BMDC, cdc42 activation assay shown in Figure 4A, western blot shown in Figure 1B and Additional file 3: Figure S3, immunofluorescence microscopy shown in Figure 4C, and data analyses. Junko Iijima carried out western blot shown in Figure 1A, 2B, and Additional file 1: Figure S1, and statistical analysis for Figure 2C, and quantitative real-time PCR shown in Figure 2A. Mitsuo Maruyama participated in the design and coordination of the study, and helped to draft the manuscript.

## Supplementary Material

Additional file 1 **Figure S1**. Equal amount of protein loaded to the gel. The proteins on the membrane were stained with pomso. Click here for file

Additional file 2 **Figure S2**. Over-expression of Cdc42 induced filopodial formation. 293T cells transfected with empty vector, or expression vectors with Cdc42WT, Cdc42V12, or Cdc42N17 were stained with phalloidin (left panel) and anti-Cdc42 antibody (middle panel) followed by immunofluorescence microscopy. Right panels are merged images of phalloidin-stained and anti-Cdc42-stained images. White bars are 10 μm. Click here for file

Additional file 3 **Figure S3**. Inhibition of degradation partially involved in up-regulation of Zizimin2 in response to stimulation. 10^7^cells/ml of BMDC with 1 h treatment of 10 μM MG132 or without the treatment that were stimulated with 1 μg/ml anti-IgG Fab'2 for 2 min (left panel, anti-FcγRII/III) or 1 μg/ml LPS for 5 min (right panel) were lysed and subjected to western blotting. α-Tubulin was used as a loading control. Click here for file
