# Supplementary figures and images for: Age-related guanine nucleotide exchange factor, mouse Zizimin2, induces filopodia in bone marrow-derived dendritic cells
Source: Immun Ageing. 2012 Apr 11;9:2. doi: 10.1186/1742-4933-9-2 (PMC3359169; doi:10.1186/1742-4933-9-2)

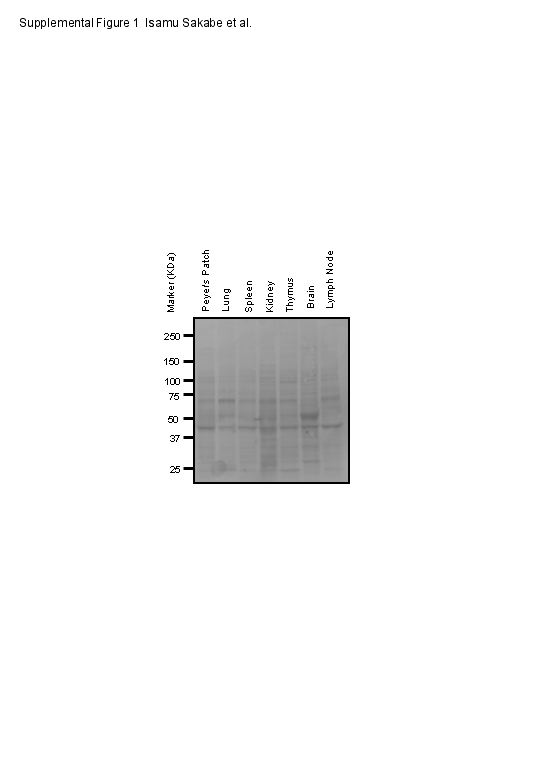

Supplement: Additional file 1 — Figure S1. Equal amount of protein loaded to the gel. The proteins on the membrane were stained with pomso. [file 1742-4933-9-2-S1.JPEG]

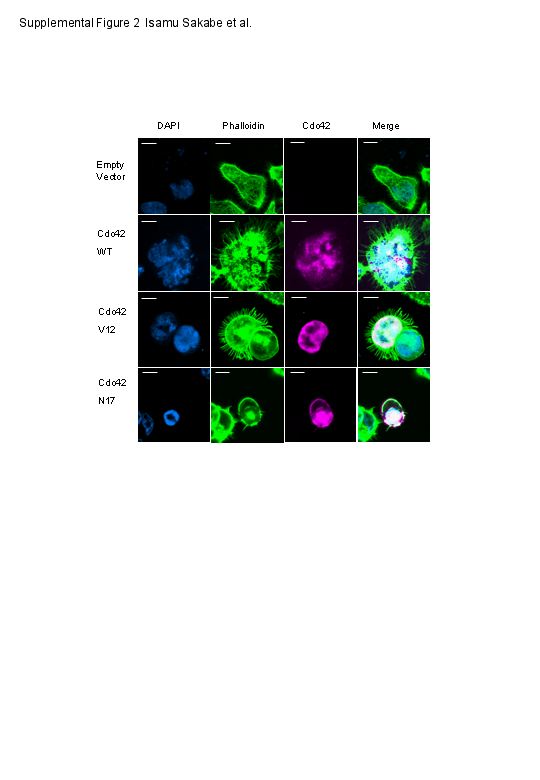

Supplement: Additional file 2 — Figure S2. Over-expression of Cdc42 induced filopodial formation. 293T cells transfected with empty vector, or expression vectors with Cdc42WT, Cdc42V12, or Cdc42N17 were stained with phalloidin (left panel) and anti-Cdc42 antibody (middle panel) followed by immunofluorescence microscopy. Right panels are merged images of phalloidin-stained and anti-Cdc42-stained images. White bars are 10 μm. [file 1742-4933-9-2-S2.JPEG]

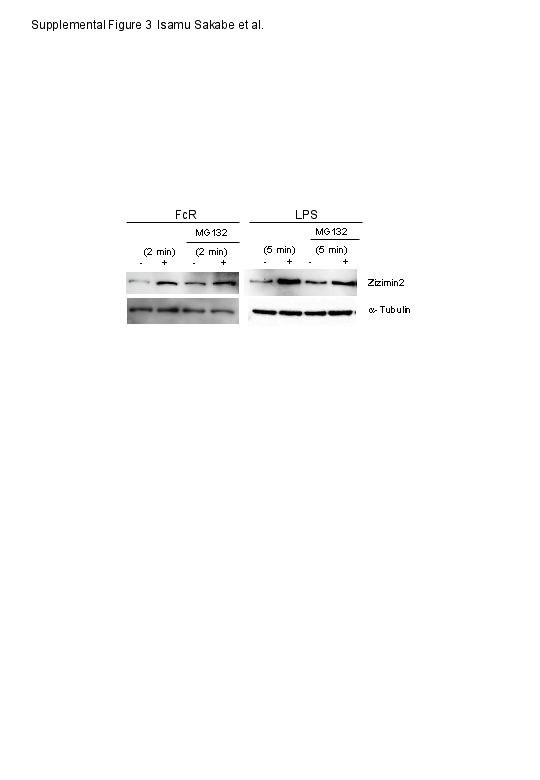

Supplement: Additional file 3 — Figure S3. Inhibition of degradation partially involved in up-regulation of Zizimin2 in response to stimulation. 107cells/ml of BMDC with 1 h treatment of 10 μM MG132 or without the treatment that were stimulated with 1 μg/ml anti-IgG Fab'2 for 2 min (left panel, anti-FcγRII/III) or 1 μg/ml LPS for 5 min (right panel) were lysed and subjected to western blotting. α-Tubulin was used as a loading control. [file 1742-4933-9-2-S3.JPEG]
